# *In vitro* performance and* in vivo* fertility of antibiotic-free preserved boar semen stored at 5 °C

**DOI:** 10.1186/s40104-020-00530-6

**Published:** 2021-01-11

**Authors:** Helen Jäkel, Kathi Scheinpflug, Kristin Mühldorfer, Rafael Gianluppi, Matheus Schardong Lucca, Ana Paula Gonçalves Mellagi, Fernando Pandolfo Bortolozzo, Dagmar Waberski

**Affiliations:** 1 Unit of Reproductive Medicine of the Clinics/Clinic for Pigs and Small Ruminants, University of Veterinary Medicine Hannover, Bünteweg 15, 30559 Hannover, Germany; 2 Department of Wildlife Diseases, Leibniz Institute for Zoo and Wildlife Research, Alfred-Kowalke-Straße 17, 10315 Berlin, Germany; 3 Animal Science Department, Swine Sector, Federal University of Rio Grande do Sul, Avenida Bento Gonçalves, 9090, Porto Alegre, 91540-000 Brazil

**Keywords:** Antibiotics, Bacteria, Boar semen, Chilling, Fertility, Semen preservation

## Abstract

**Background:**

Hypothermic preservation of boar semen is considered a potential method for omitting antibiotics from insemination doses, thereby contributing to the global antibiotic resistance defence strategy. The main challenges are chilling injury to spermatozoa and bacterial growth during semen storage leading to reduced fertility.

**Objectives:**

To examine chilling injury and the number and type of bacteria in boar semen stored at 5 °C in the absence of antibiotics, and to assess the applicability of hypothermic semen storage under field conditions.

**Material and methods:**

Boar ejaculates were extended with AndroStar® Premium, stored at 17 °C with and at 5 °C without antibiotics and tested for functional sperm parameters by flow cytometry. Raw semen and extended samples were investigated bacteriologically. Fertility was evaluated after once-daily inseminations of 194 sows in a field study.

**Results:**

Lethal sperm damage assessed by motility and membrane integrity was low throughout storage in both experimental groups. Sublethal chilling effects based on the decrease of viable spermatozoa with low membrane fluidity were higher (*P* < 0.05) up until 72 h in sperm stored at 5 °C compared to 17 °C but did not differ after 144 h. After 72 h, incubation in capacitating medium for 60 min induced a similar decrease in viable sperm with high mitochondria membrane potential and low cytosolic calcium in both groups. In semen stored at 5 °C, bacteria counts were below 10^3^ CFU/mL and the bacteria spectrum was similar to that of raw semen. In 88% of 34 boars, cooled semen fulfilled the requirements for insemination. Fertility was high and did not differ (*P* > 0.05) between sow groups inseminated with semen stored antibiotic-free at 5 °C and semen stored at 17 °C with antibiotics.

**Conclusion:**

Despite subtle chilling effects and low bacterial numbers, antibiotic-free hypothermic storage of boar semen offers the possibility to reduce the use of antibiotics in pig insemination. However, strict sanitary guidelines must be maintained and further evidence of efficiency under field conditions is considered desirable.

**Supplementary Information:**

The online version contains supplementary material available at 10.1186/s40104-020-00530-6.

## Background

Semen of genetically superior boars are used for artificial insemination (AI) as an efficient method to minimise the risk of transmitting venereal diseases and to accelerate genetic progress. Introducing this assisted reproductive technology has been successful worldwide, being used in more than 90% of sows on breeding farms [1]. Control of microbial growth is ensured by routinely adding antibiotics to semen extenders, which is currently mandatory in the European Union [2]. With this, the growth of bacteria, most of them originating as natural commensals from the male’s genital tract, can be reduced during liquid storage of AI doses. Similar to other fields with permanent antibiotic use, however, this practice favours the emergence, spread and persistence of multidrug-resistant bacteria. Awareness of antibiotic resistance as a global threat to human, animal and environmental health [3] has stimulated the search for alternative strategies in artificial breeding in livestock [1, 4]. The main challenge is realising an efficient action of any alternative antimicrobial concept against bacteria without being harmful to sperm.

Only recently was the hypothermic semen storage at 5 °C in the absence of antibiotics proposed as an alternative to the common storage at 17 °C with antibiotics [5]. The underlying concept relies on low-temperature storage to act bacteriostatically in order to maintain bacterial load below critical thresholds [5, 6], as exceedance thereof is detrimental to sperm quality and might affect the female genital tract [7–11].

However, a major concern regarding the 5 °C storage concept is the high sensitivity of boar spermatozoa to chilling injury. This is due to the high content of polyunsaturated fatty acids and low sterol to phospholipid ratio in the sperm membranes [12]. Despite the encouraging *in vitro* results and high fertility in a first large-scale field study [5] confirmed by a follow-up *in vitro* study [6], more in-depth knowledge on potential sublethal sperm damage induced by chilling and storage stress is needed. Cold shock results in lethal damage to a minor sperm population, but, more importantly, leads to major sublethal damage [13], compromising functional sperm parameters which remain undetected by conventional semen analyses [13, 14]. Multiparametric flow cytometry now opens new possibilities for the simultaneous assessment of multiple cellular compartments and functions within the same spermatozoon [15], thus providing benefits in the overall assessment of sperm fertilising capacity [16].

It is known that boars differ in the tolerance of their sperm to chilling or freezing [17]. To date, information about the ratio of AI boars regarding their eligibility for successful hypothermic semen storage is unknown. In addition to cooling-induced cell death as commonly assessed by loss of motility and/or membrane integrity, viable sperm could be functionally affected. Sperm chilling stress alters the dose-response for herd fertility towards a higher sperm number in AI doses in order to reach the plateau of the asymptotic fertility curve [18], mostly by affecting the sperm transit through the female tract and the interaction with female tissue and local immune cells [19]. Hence, sensitive characterisation of sperm functionality and *in vivo* inseminations in different AI management settings are required to assess the potential of hypothermic semen storage for replacing antibiotics in boar semen extenders.

In the light of this background, the aims of this study were first, to assess chilling-induced sublethal damage on hypothermic stored boar spermatozoa by multicolour flow cytometry and second, to assess the fertility of antibiotic-free cold-stored semen under well standardised field conditions. Moreover, the number and type of bacteria during prolonged storage of up to 72 h at 5 °C in the absence of antibiotics were monitored.

## Materials and methods

### Chemicals and media

Chemicals were purchased from Sigma Aldrich (Steinheim, Germany) and Carl Roth (Karlsruhe, Germany) if not otherwise indicated and were of analytical grade. The fluorochromes Calbryte 630 and Merocyanine 540 (M540) were obtained from Biomol (Hamburg, Germany). Hoechst 33342 (H 33342), Hoechst 33258 (H 33258), Peanut Agglutinin (PNA)-Alexa-647 and 4-[(3-methyl-1,3-benzoxazol-2(3H)-ylidene)methyl]-1-[3-(trimethyl-ammonio)propyl]-quinolinium diiodide (Yo-Pro 1) were obtained from Life Technologies (Thermo Fisher, Darmstadt, Germany). The Silicon rhodamine fluorophore analogue (SiR700)-DNA was purchased from tebu-bio (Offenbach am Main, Germany). The dye 5,5′,6,6′-tetrachloro-1,1′,3,3′-tetraethylbenzimidazolocarbocyanine iodide (JC-1) was purchased from Enzo Life Science (Lörrach, Germany). Semen extenders were obtained from Minitüb (Tiefenbach, Germany).

The capacitating medium Tyrode A for calcium influx experiments was made of 96 mmol/L NaCl, 3.1 mmol/L KCl, 0.4 mmol/L MgSO_4_, 5 mmol/L glucose, 15 mmol/L NaHCO_3_, 2 mmol/L CaCl_2_, 0.3 mmol/L KH_2_PO_4_, 20 mmol/L 4-(2-hydroxyethyl)-1-piperazineethane-sulfonic acid (HEPES), 21.7 mmol/L sodium lactate, 1.0 mmol/L sodium pyruvate, 100 μg/mL gentamicin (SERVA, Heidelberg, Germany), 20 μg/mL phenol red, 3 mg/mL bovine serum albumin (BSA). The pH was corrected with NaOH to 7.6 at 20 °C and osmolarity was adjusted with NaCl to 295–305 mOsmol/kg. For equilibration, the medium was incubated in a cabinet with 5% CO_2_ at 38 °C. Tyrode’s medium C (control) lacked the capacitation inducer NaHCO_3_. The pH was corrected with NaOH to 7.55 at 20 °C and osmolarity was adjusted with NaCl to 295–305 mOsmol/kg. The medium was incubated in a heating cabinet at 38 °C before use.

HEPES-buffered saline medium (HBS) consisted of 0.137 mol/L NaCl, 0.02 mol/L HEPES, 0.01 mol/L glucose and 0.0025 mol/L KOH. The pH was adjusted with NaOH to 7.55 at 20 °C and osmolarity was adjusted to 295–305 mOsmol/kg with NaCl.

### Experiment 1: *in vitro* assessment of chilling effects

#### Animals, semen processing and storage

Semen were collected using the “gloved hand” method once a week from nine sexually mature fertile boars (eight Piétrain and one Large White) which were housed at the Unit of Reproductive Medicine, University of Veterinary Medicine Hannover, Germany. The boars were between 12 months and 5 years of age. One ejaculate per boar was collected.

The pre-spermatic fraction was discarded and the ejaculate (sperm-rich and sperm-poor fractions) were collected in a pre-warmed thermal cup. Normospermic ejaculates (≥ 20 × 10^9^ spermatozoa, ≥ 70% total sperm motility, > 75% morphologically normal spermatozoa) were split into three aliquots. Aliquots were extended in AndroStar® Premium (APrem; Minitüb, Tiefenbach, Germany) without antibiotics (AB), APrem with AB (0.25 g/L gentamicin sulphate), or Beltsville Thawing Solution (BTS, Minitüb, Tiefenbach, Germany) with AB (0.25 g/L gentamicin sulphate). The BTS-diluted semen was used as a control for microbiological investigations to provide a semen dose that is commonly used for AI. Extended semen doses had a final concentration of 20 × 10^6^ sperm/mL in 90 mL flexitubes, resulting in a total of 1.8 × 10^9^ sperm. A separate tube was produced for each analysis day. The tubes were stored in two separate cardboard boxes, one for each storage temperature. Each box contained a total of 35 tubes and was kept for 2 h at room temperature (21 °C). The box with the samples containing AB were subsequently stored in a 17 °C storage cabinet in the dark for a total of 144 h. The box with the samples lacking antibiotics was slowly cooled and stored in a temperature-controlled 5 °C storage cabinet. Cooling velocities were in accordance with the suggested ideal cooling rates as previously shown [20].

#### Acrosome assessment

Aliquots of semen samples were fixed in 300 μL formol citrate. A phase contrast microscope (Zeiss, Oberkochen, Germany) and 1000 × magnification with oil immersion was used. A total of 200 spermatozoa per sample were analysed for acrosome integrity after 24 h, 72 h and 144 h of semen storage.

#### Computer-assisted semen analysis (CASA)

Sperm motility was assessed after 24 h, 72 h and 144 h using the CASA system AndroVision®, version 1.1.6. (Minitüb, Tiefenbach, Germany) and a phase contrast microscope (Zeiss, Oberkochen, Germany) equipped with a heated stage, a 10 × ocular and a 20 × objective. Aliquots of semen samples were incubated for 30 min at 37 °C before being transferred to pre-heated 20 μm deep Leja chambers (Leja, Nieuw Vennep, The Netherlands). A minimum of 600 sperm cells were analysed per sample within five centrally located chamber fields, each with a rate of 60 frames per second. Total motile spermatozoa were defined as having a curvilinear velocity (VCL) > 24 μm/s and an average amplitude of lateral head displacement (ALH) > 1 μm.

#### Flow cytometry

Flow cytometry with the combined use of four stains was conducted using a CytoFlex flow cytometer (Beckman Coulter, Krefeld, Germany) equipped with three lasers of wavelengths 405, 488 and 638 nm.

Membrane integrity and fluidity were assessed simultaneously after 24 h, 72 h and 144 h of storage. Samples with 480 μL of pre-incubated extended semen (15 min, 38 °C) were stained with Hoechst 33342 (final concentration: 0.9 μg/mL), Yo-Pro 1 (final concentration: 0.02 μmol/L), Merocyanine 540 (final concentration: 0.54 μmol/L) and PNA-Alexa-647 (PNA; final concentration: 1 μg/mL) and co-incubated for 15 min at 38 °C in the dark. Then, aliquots of 50 μL were transferred to 950 μL HBS and a total of 10,000 events were assessed by CytExpert software (version 3.2, Beckman Coulter, Krefeld, Germany). Signals were detected using filters 450/45 BP (H 33342), 525/40 BP (Yo-Pro 1), 780/60 BP (PNA-Alexa-647) and 585/42 BP (M540). Viable spermatozoa were defined as plasma membrane intact spermatozoa (H 33342 positive, Yo-Pro 1 negative) and further characterised for acrosome integrity (PNA-Alexa-647 negative) and membrane fluidity (M540 negative or positive).

In a second approach, intracellular calcium content and mitochondrial membrane potential (MMP) were assessed simultaneously after 72 h of storage. Aliquots of the diluted samples were stained with Calbryte 630 (final concentration: 0.91 μmol/L) and incubated for 60 min at 38 °C in the dark. The fluorochromes SiR700-DNA (final concentration: 0.014 μmol/L), Hoechst 33258 (final concentration: 0.27 μg/mL) and JC-1 (final concentration: 0.56 μmol/L) were added and co-incubated for 15 min. A volume of 50 μL of stained sample was then transferred to 950 μL pre-warmed capacitating medium (Tyrode A) and non-capacitating control medium (Tyrode C), followed by incubation at 38 °C in CO_2_ (5% saturation, 100% humidity; Tyrode A) or in air (control). After incubation for 3 and 60 min, 10,000 events were assessed by flow cytometry. Signals were detected using filters 450/45 BP (H 33258), 525/40 BP (JC-1 monomer), 585/42 BP [JC-1 aggregate (agg)], 660/20 BP (Calbryte 630) and 712/25 BP (SiR700-DNA). Viable spermatozoa were defined as plasma membrane intact spermatozoa (SiR700-DNA positive, H 33258 negative) and further characterised for intracellular calcium levels by Calbryte 630. The MMP for viable sperm was detected by JC-1 staining.

#### Microbiology

Raw semen from the nine boars in experiment 1 and the corresponding split semen samples (extended with AB-free APrem or BTS with AB) stored for 0 h, 24 h, 48 h and 72 h were transferred from separate tubes to sterile cryo vials with a final concentration of 20% glycerol to protect bacteria cells from freeze-thaw damage, and then shock-frozen and stored in liquid nitrogen. Frozen samples were shipped on dry ice within 6 h to the Leibniz Institute for Zoo and Wildlife Research, Berlin, Germany and stored at − 80 °C for microbiological investigations.

The total bacterial cell count in extended semen samples was determined from a ten-fold serial dilution prepared in PBS ranging from 10^− 1^ to 10^− 3^. A volume of 100 μL of each dilution was plated on LB agar plates (Carl Roth, Karlsruhe, Germany) and incubated for 48 h at 37 °C under aerobic conditions. Bacterial numbers were calculated from two dilutions in duplicate and expressed as colony-forming units per millilitre (CFU/mL).

For bacterial isolations, 10 μL of the extended semen samples with and without AB were plated onto a plate set routinely used for microbial diagnostics [Columbia agar with 5% sheep blood, Gassners’ agar and UTI Clarity agar (Oxoid Thermo Fisher, Wesel, Germany)]. After aerobic incubation for 24 h at 37 °C, the initial blood agar plates were re-incubated once more for 24 h with 5% CO_2_ and analysed for slow and fastidious growing species. On both occasions, distinct colonies were subcultured on the same plate set and incubated for another 24 h under the same conditions. Bacterial identification from pure subcultures followed standard diagnostic procedures and was accomplished by classical biochemical tests including the API® identification system from bioMérieux Deutschland (Nürtingen, Germany) or 16S rDNA gene analysis as described by Mühldorfer et al. [21]. The different subcultures were finally assigned to three main groups (Gram-positive cocci, Gram-positive rods or Gram-negative bacteria) for comparison because of the diversity of bacterial species and isolates that could not be classified to genus or species levels. Among those, bacterial isolates of potential concern for AI were recorded separately. This category included species that could negatively affect spermatozoa or bear specific risks in semen production [22], such as potential extended spectrum beta-lactamase (ESBL) or biofilm-producing bacteria, as well as species that can cause bacterial infections in sows.

### Experiment 2: fertility *in vivo* under field conditions

#### Boars, semen processing and storage

Ejaculates of 34 fertile boars (Agroceres AG337 PIC®, Patos de Minas, MG, Brazil) housed in an AI centre in Brazil (Master Agroindustrial) were tested for their eligibility for hypothermic semen storage. Entire ejaculates (except for the pre-spermatic fraction) were collected manually with the “gloved-hand” method. Normospermic ejaculates (one ejaculate per boar) were split into two aliquots and diluted to a concentration of 30 × 10^6^ sperm/mL, resulting in 1.5 × 10^9^ sperm per 50 mL dose with APrem with AB (0.25 g/L gentamicin) and without AB. Separate semen tubes were produced for each analysis day. Samples diluted with AB were stored in a plastic bag, then hold for 90 min at room temperature (21 °C) and subsequently stored in a 17 °C storage cabinet. Package and cooling conditions were adapted to the AI-lab conditions to ensure that cooling rates were in the optimal range [20] and corresponded to the cooling rates used in experiment 1. For this, samples diluted without AB were stored together with a total of 37 isothermic tubes in a closed cardboard box lined with a double layer of bubble wrap on the inside, hold for 1.5–3 h at room temperature (21 °C) and then stored in a temperature-controlled 5 °C storage cabinet. All samples were stored for a total of 120 h.

Randomly selected normospermic ejaculates of 15 boars with satisfactory storage results after 120 h of low temperature storage (≥ 65% total motility and acrosome integrity ≥85%) were prepared for the insemination trial. A total of six semen pools with different combinations of boars (four ejaculates/pool) were produced once a week (two pools per day) over a three-week period. Semen pools were split and isothermically diluted in APrem without AB and APrem with AB (0.25 g/L gentamicin sulphate) to a final concentration of 50 × 10^6^ sperm/mL, resulting in 2.5 × 10^9^ sperm per 50 mL dose. Semen doses were cooled and stored as described above.

#### Spermatology

Sperm motility was assessed after 24 h, 72 h and 120 h using the CASA system AndroVision®, version 1.2. (Minitüb, Tiefenbach, Germany) with the identical method and settings as described in experiment 1, using a phase contrast microscope (Olympus, Tokyo, Japan). Acrosome integrity was assessed after 24 h, 72 h and 120 h using a phase contrast microscope (Olympus, Tokyo, Japan) as described in experiment 1.

#### Insemination and fertility recording

On a farm located one mile from the boar stud, 416 sows (Camborough Agroceres PIC®, Patos de Minas, Brazil) were available for insemination during the three-week period. At weaning, sows (*n* = 332) were selected according to the following parameters: parity 1 to 7, number of total born piglets not fewer than five piglets below herd average (15.1 ± 0.2) in the last litter, lactation length of 17–23 d and body condition score (BCS) of 2–3.5 [23]. Oestrus detection was conducted once in the morning by boar activity. Standing reflex was confirmed by applying pressure on the back of the sow. Only sows with a weaning-to-oestrus interval of 4 d (*n* = 206) were considered for standardising the insemination-ovulation interval and storage duration of semen tubes. Selected sows were randomised according to the above mentioned parameters ordered by parity, total born piglets and BCS to form two insemination groups for the 5 °C- and 17 °C-stored semen, respectively (Table 1). Sows (*n* = 194) were inseminated post-cervically once a day directly after the first oestrus detection until the end of oestrus with a 24-h interval, resulting in two to three (averages: 17 °C, 2.55; 5 °C, 2.52) inseminations per oestrus. In both groups, first insemination was performed with semen stored for 24 h and the second insemination with semen stored for 48 h. In case of a third insemination, semen stored for 72 h were used. All inseminations per oestrus were performed with semen from the same pool. Twelve eligible sows were not considered for insemination to avoid numeric imbalance between the weekly insemination groups.

**Table 1:** Means ± SEM in the two insemination groups for parameters used for randomisation (experiment 2)

Semen storage	Sows, *n*	Parity order	Total born piglets, *n*^**a**^	Body condition score	Total weaned piglets, *n*^**a**^	Lactation length, d^**a**^
17 °C w/ AB	97	2.74 ± 0.16	15.55 ± 0.27	2.79 ± 0.03	12.81 ± 0.13	19.92 ± 0.13
5 °C w/o AB	97	2.76 ± 0.15	15.52 ± 0.28	2.80 ± 0.04	12.85 ± 0.15	19.93 ± 0.13

^a^from the last litter preceding the insemination trial

17 °C: semen stored in AndroStar® Premium with antibiotics (w/AB; 0.25 g/L gentamicin sulphate)

5 °C: semen stored in AndroStar® Premium without antibiotics (w/o AB)

Fertility parameters were monitored with return-to-oestrus checks by boar detection starting at d 17 after the first insemination. Pregnancy checks were performed with transabdominal ultrasound at d 24 after insemination. Farrowing rates and the total number of born, live-born, stillborn and mummified piglets were recorded. Five sows were excluded (one in the 17 °C-group and four in the 5 °C-group) from the evaluation due to disease or death not related to the reproductive tract.

### Statistical analysis

Statistical data analysis was conducted with CytExpert 2.3, Excel, Kaluza Analysis 2.1, and SAS Enterprise Guide, version 7.1. Data were tested for normal distribution (Shapiro-Wilk test) followed by pairwise comparisons using one-way ANOVA and two-way ANOVA mixed models (PROC MIXED) for repeated measurements. Data that were not normally distributed were analysed with Wilcoxon’s Signed Rank test (PROC UNIVARIATE). Unpaired data sets were analysed using the Fisher-exact test (PROC FREQ), Chi^2^-test and nonparametric one-way ANOVA (PROC NPAR1WAY). Data were compared for storage temperature differences and then for storage time points, if applicable. A value of *P* < 0.05 was considered as statistically significant. Data are shown as means ± standard error of the mean (SEM).

## Results

### Experiment 1: *in vitro* assessment of chilling effects

#### Sperm motility

**Fig. 1:**
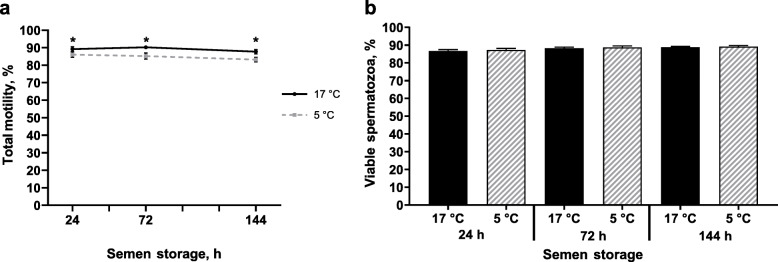
Lethal sperm damage in semen samples preserved in AndroStar® Premium extender and stored at 17 °C with antibiotics (0.25 g/L gentamicin sulphate) or at 5 °C without antibiotics (*n* = 9 boars, experiment 1). **a** Total motile spermatozoa (%; means ± SEM). *Values differ between storage temperature groups at a given time point (*P* < 0.05). **b** Viable (Yo-Pro-1 negative) spermatozoa (%; means ± SEM). Values do not differ between storage temperature groups

Total motility remained on a high level throughout storage with slightly higher values in samples stored at 17 °C compared to 5 °C (*P* < 0.05; Fig. 1a). Total motility ranged from 89.2% ± 1.3% (24 h) to 87.7% ± 1.3% (144 h) for 17 °C-stored samples and from 86.0% ± 1.6% at 24 h to 83.2% ± 1.3% at 144 h for 5 °C-stored samples.

#### Flow cytometry

The proportion of spermatozoa with intact plasma membranes (viability; Yo-Pro 1 negative) did not differ between the semen groups (*P* = 0.4961; Fig. 1b). Values ranged from 86.7% ± 0.8% (24 h) to 88.9% ± 0.4% (144 h) for 17 °C-stored spermatozoa and from 87.3% ± 0.9% (24 h) to 89.2% ± 0.6% (144 h) for 5 °C-stored spermatozoa.

**Fig. 2:**
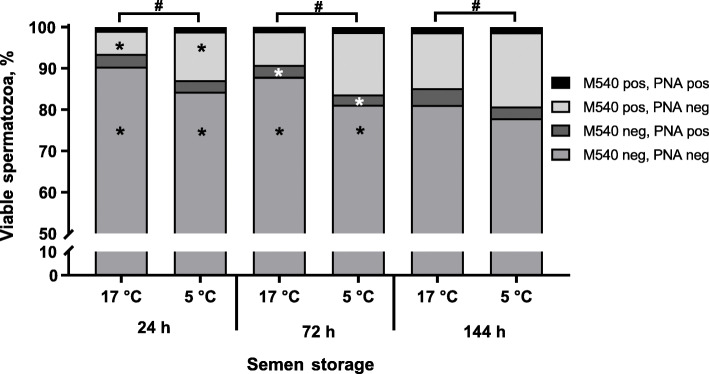
Sublethal sperm damage in semen samples preserved in AndroStar® Premium extender and stored at 17 °C with antibiotics (0.25 g/L gentamicin sulphate) or at 5 °C without antibiotics (*n* = 9 boars, experiment 1): Distribution of viable (Yo-Pro-1 neg.) spermatozoa (%) with low (M540 negative) or high (M540 positive) membrane fluidity and intact (PNA negative) or defective (PNA positive) acrosome. *Values of same sperm classes differ between storage temperature groups at a given time point (*P* < 0.05). ^#^Values for overall sperm distribution in all four classes differ between storage temperature groups at a given time point (*P* < 0.05)

Membrane fluidity and acrosome integrity in viable spermatozoa were measured and evaluated in a combined assay. Results are shown in Fig. 2 and in Table S1. The overall distribution of spermatozoa assigned to the four possible combinations of fluorescence patterns varied between temperatures at all storage times (*P* < 0.05). In both semen groups at all time points, the main population of viable spermatozoa, ranging between 90.4% ± 2.3% (17 °C, 24 h) and 77.9% ± 5.1% (5 °C, 144 h), displayed low membrane fluidity with intact acrosomes (M540 negative, PNA negative). At 24 h and 72 h, values for this sperm population were higher at 17 °C compared to 5 °C (*P* < 0.05). The second largest population consisting of spermatozoa with high membrane fluidity and intact acrosomes (M540 positive, PNA negative) varied between 5.6% ± 2.5% (17 °C, 24 h) and 18.0% ± 5.3% (5 °C, 144 h). At 24 h, values for this sperm population were lower at 17 °C compared to 5 °C (*P* < 0.05).

**Fig. 3:**
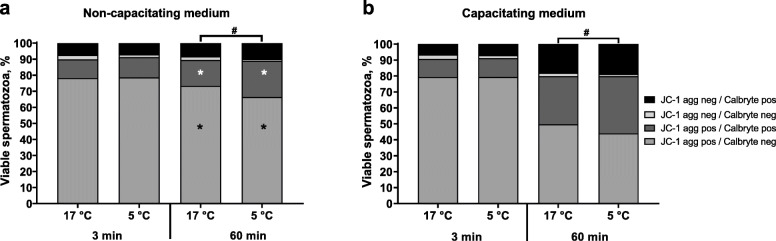
Sublethal damage of sperm function under capacitating and non-capacitating conditions in semen samples preserved in AndroStar® Premium extender and stored at 17 °C with antibiotics (0.25 g/L gentamicin sulphate) or at 5 °C without antibiotics (*n* = 9 boars, experiment 1): Distribution of viable (Hoechst 33258 negative) spermatozoa (%) with high (JC-1 aggregate positive) or low (JC-1 aggregate negative) mitochondria membrane potential and high (Calbryte 630 positive) or low (Calbryte 630 negative) intracellular calcium **a** after incubation in non-capacitating medium (Tyrode C) and **b** after incubation in capacitating medium (Tyrode A). *Values of same sperm classes differ between storage temperature groups at a given time point (*P* < 0.05). ^#^Values for overall sperm distribution in all four classes differ between storage temperature groups at a given time point (*P* < 0.05)

After semen storage for 72 h, intracellular calcium content and MMP in viable spermatozoa were measured and evaluated in a combined assay under capacitating and control conditions. Results are shown in Fig. 3 and in Table S2. The main population for all incubation conditions consisted of spermatozoa with high MMP and low intracellular calcium content (JC-1 agg pos./Calbryte neg.) and the proportion of sperm in this population did not differ (*P* = 0.7344) between the storage temperature groups at the onset of incubation (3 min). During incubation in the control medium, this population decreased moderately from 78.2% ± 0.9% to 73.3% ± 2.0% (17 °C, *P* < 0.05) and from 78.6% ± 1.1% to 66.3% ± 2.5% (5 °C, *P* < 0.05). After 60 min incubation in capacitating medium, the population decreased for 17 °C-stored spermatozoa from 79.3% ± 1.2% to 49.6% ± 5.6% (*P* < 0.05), and for 5 °C-stored spermatozoa from 79.3% ± 1.0% to 44.0% ± 3.9% (*P* < 0.05).

The second largest sperm population consisted of spermatozoa with high MMP and high intracellular calcium (JC-1 agg pos./Calbryte pos.). The proportion of sperm in this population did not differ between the storage temperature groups at the onset of incubation (3 min). After incubation in the control medium, this population was higher in semen stored at 5 °C compared to 17 °C (*P* < 0.05). Under capacitating conditions, the sperm population increased for both storage temperature groups similarly from 11.3% ± 1.0% to 30.3% ± 4.0% (17 °C, *P* < 0.05) and from 11.9% ± 0.6% to 35.8% ± 2.5% (5 °C, *P* < 0.05), respectively.

#### Microbiology

**Fig. 4:**
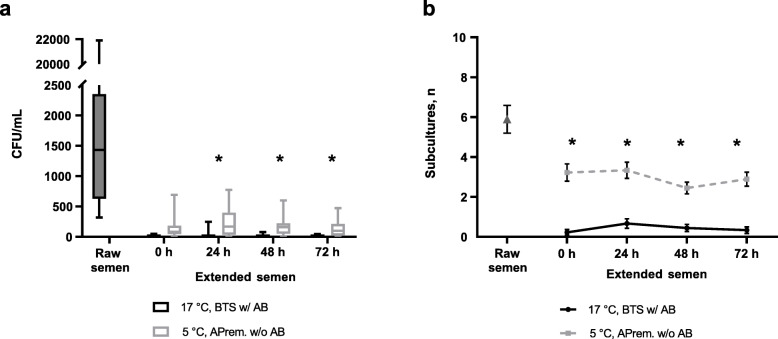
Presence of bacteria in raw semen and in semen samples preserved in BTS extender with antibiotics (0.25 g/L gentamicin sulphate; BTS w/AB) at 17 °C or in AndroStar® Premium extender without antibiotics (APrem w/o AB) at 5 °C (*n* = 9 boars, experiment 1). **a** Box-whisker plots showing bacterial counts (CFU/mL). **b** Numbers of subcultures detected during storage. *Values differ between storage temperature groups at a given time point

**Fig. 5:**
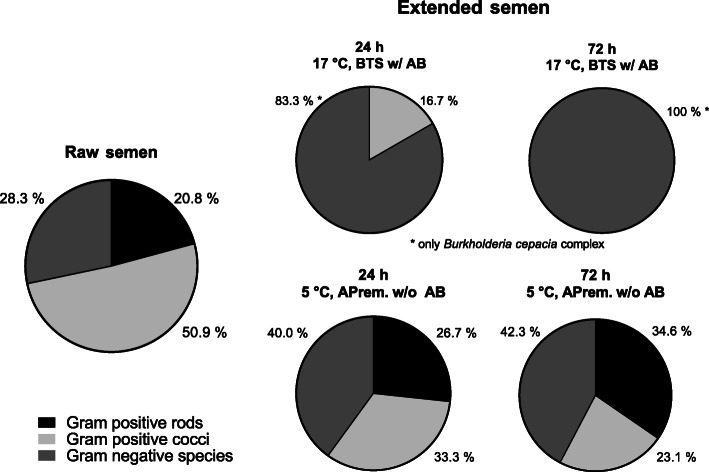
Bacteria species isolated from raw semen and from split semen samples preserved in BTS extender with antibiotics (0.25 g/L gentamicin sulphate; BTS w/AB) at 17 °C or in AndroStar® Premium extender without antibiotics (APrem w/o AB) at 5 °C (*n* = 9 boars, experiment 1)

Bacterial counts of semen samples from nine boars are presented in Fig. 4a. In raw semen samples, 3647 ± 2296 CFU/mL (range 318 to 21,900 CFU/mL) were determined. During storage for 72 h, bacterial counts ranged between 0 and 245 CFU/mL in semen samples extended in BTS with AB at 17 °C, and between 0 and 773 CFU/mL in samples extended in APrem stored at 5 °C without AB. Bacterial numbers in extended semen decreased between 24 and 72 h from 30 ± 27.0 to 5 ± 5.1 CFU/mL in 17 °C-stored BTS samples (with AB) and from 244 ± 84.4 to 134 ± 51.7 CFU/mL in 5 °C-stored APrem samples without AB. At all time points, bacterial counts were higher in AB-free samples stored at 5 °C compared to samples stored at 17 °C with AB (*P* < 0.05). Raw semen contained on average 5.9 ± 0.7 distinct subcultures (Fig. 4b). The average number of subcultures remained constant during a storage of 72 h with higher numbers in 5 °C-stored samples (3.0 ± 0.2) compared to 17 °C-stored samples (0.4 ± 0.1; *P* < 0.05). In raw semen samples, 50.9% of the subcultures were identified as Gram-positive cocci, 28.3% as Gram-negative species (mainly non-fermenting bacteria) and 20.8% as Gram-positive rods (Fig. 5). The spectrum remained similar in the APrem AB-free extended samples at 5 °C but showed a constant decrease in the Gram-positive cocci by half until 72 h of storage. In contrast, samples stored in BTS with AB at 17 °C showed a shift towards Gram-negative species already after 24 h (83.3%) to 100% after 72 h of storage. The Gram-negative species were identified as bacteria belonging to the *Burkholderia cepacia* complex and were only isolated from diluted samples but not from raw semen. Three other bacterial species of potential concern in AI, namely *Pseudomonas aeruginosa*, *Pasteurella* sp. and *Escherichia coli*, were isolated from raw semen and from the samples extended in APrem without AB as summarised in Table S3. Likewise, *B. cepacia, P. aeruginosa* (*n* = 6) were isolated at 72 h of storage at 5 °C, meanwhile *Pasteurella* (*n* = 3) was found sporadically at 24 h of storage and *E. coli* (*n* = 1) only initially (0 h).

### Experiment 2: fertility *in vivo* under field conditions

#### Spermatology

**Fig. 6:**
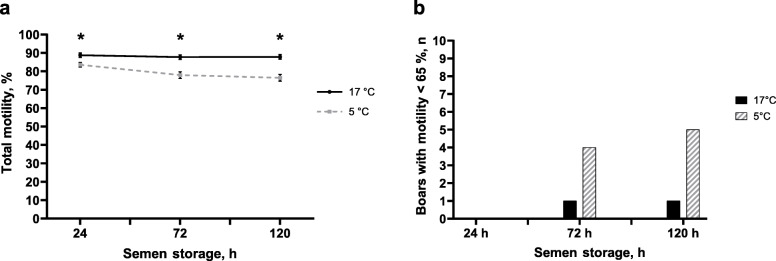
Sperm motility of boars (*n* = 34) in an AI centre (experiment 2). Split semen samples were preserved in AndroStar® Premium extender and stored at 17 °C with antibiotics or at 5 °C without antibiotics. **a** Total motile spermatozoa (%; means ± SEM). *Values differ between storage temperature groups at a given time point (*P* < 0.05). **b** Boars (*n*) with sperm motility less than 65% in both semen groups

In 34 boars at the AI centre, sperm motility was higher in samples stored at 17 °C compared to 5 °C at all time points (*P* < 0.05; Fig. 6). For 17 °C-samples, motility at 24 h was 88.7% ± 1.2% and at 120 h it was 87.8% ± 1.3% (*P* > 0.05). Values for 5 °C-stored samples were 83.6% ± 1.2% at 24 h, these declining to 76.5% ± 1.8% at 120 h (*P* < 0.05). Thresholds for usable semen for AI were defined as > 65% total motility [1]. After 72 and 120 h of storage, one sample (2.9%) for 17 °C-stored semen did not meet the threshold requirements, whereas for 5 °C-stored semen, four samples (11.8%) at 72 h and five samples (14.7%) at 120 h failed to meet these requirements. The proportion of spermatozoa with damaged acrosomes was less than 10% in all samples at any time point of storage.

Total motility for pooled semen used for AI remained > 88.5% during storage (120 h) for both temperature groups. Samples stored at 17 °C showed higher sperm motility at 72 h (17 °C: 93.0% ± 0.9%, 5 °C: 88.5% ± 1.0%; *P* < 0.05) and at 120 h of storage (17 °C: 93.0% ± 0.5%, 5 °C: 88.9% ± 0.6%; *P* < 0.05). Motility did not differ between time points within a given storage temperature group. Spermatozoa with defective acrosomes were fewer than 10% in all samples at any time point of storage. Sperm data are presented in Table S4.

#### Fertility results

Fertility data were at high level and did not differ between the two insemination groups (Table 2). There were 0.6 ± 0.1 stillborn piglets and 0.4 ± 0.1 mummified foetuses in the 17 °C insemination group, and 0.7 ± 0.1 stillborn piglets and 0.5 ± 0.1 mummified foetuses in the 5 °C group (*P* > 0.05).

**Table 2:** Fertility data of sows (experiment 2)

Semen storage	17 °C w/AB	5 °C w/o AB	***P***-value
Sows, *n*	94	95	
Pregnancy rate day 24, %	98.9	98.9	1.00
Farrowing rate, %	96.8	98.9	0.37
Total number of born piglets	15.3 ± 0.4	15.8 ± 0.3	0.51
Total number of live-born piglets	14.3 ± 0.4	14.6 ± 0.3	0.94

Data for total number of born and live-born piglets are shown as means ± SEM. Values do not differ (*P* > 0.05).

Sows (*n* = 189) were inseminated with semen pools extended split-sample in AndroStar® Premium and stored at 17 °C with antibiotics (17 °C w/AB; 0.25 g/L gentamicin sulphate) or at 5 °C without antibiotics (5 °C w/o AB).

## Discussion

The present study provides evidence that despite minor sublethal alterations induced by chilling, AB-free hypothermic preservation of boar semen seems to be appropriate for use in the majority of ejaculates without affecting fertility.

Potential sublethal damage was analysed with multiparametric flow cytometry, a technique that provides the opportunity to identify a multitude of sperm quality data on a single cell basis [24]. Changes in plasma membrane fluidity (also referred to as phospholipid disorder) of viable sperm with an intact acrosome were detected, thus excluding sperm which had undergone advanced capacitation-like alterations of sperm membranes with an incipient loss of acrosome integrity. Compared to conventional preservation at 17 °C, storage at 5 °C induced a shift towards a higher population of membrane-intact sperm with higher membrane fluidity, which is in agreement with previous observations [13, 25]. Interestingly, storage at 5 °C for 144 h did not result in additional major basal changes in membrane fluidity compared to 17 °C, indicating that the lipid membrane domains of sperm surviving the initial chilling stress are relatively resistant to subsequent storage stress.

Nonetheless, our data support the view [13, 26] that chilling, storage and rewarming involve the rearrangement of lipid membrane domains, resulting in an increase in membrane permeability and thus influx of extracellular calcium and probably other ions. Combined assessment of MMP and intracellular calcium levels in viable sperm demonstrates that spermatozoa with increased cytosolic calcium content are still able to maintain a high MMP. Since the maintenance of a high MMP relies on the impermeability of the mitochondrial inner membrane for protons [27], cooling, storage and rewarming of spermatozoa apparently did not affect this functional barrier. Integrity of this barrier is essential for cell viability and ATP synthesis. Sperm functionality tests under capacitating conditions led to a similar increase in the sperm subpopulation with high MMP and high calcium in both storage groups, indicating that the dynamics of mitochondrial activity required for capacitation [28] are not disturbed by the hypothermic preservation method employed during these studies.

In agreement with our previous study [5], lethal sperm damage expressed as loss of plasma membrane integrity and/or motility was low in stored semen samples extended with AndroStar® Premium and after controlled cooling to 5 °C. Resilience to semen handling and cooling stress differs between boars [17, 29] and “good” and “bad” coolers are likely to exist analogous to a similar classification of freezing tolerance, despite the fact that cryopreservation includes more stressors like centrifugation and changes of media. In the present study, screening of semen quality in an AI boar population demonstrated that 88.2% of the ejaculates from 34 different boars fulfilled minimum standards for usable semen, i.e. at least 65% motility and less than 25% abnormal sperm at 72 h of storage. From the perspective of sperm tolerance to cooling, utilising the 5 °C storage concept on a large scale appears feasible and could be facilitated if markers become available to identify the hypothermic storage ability of semen from individual boars before introducing them to the AI centre. Additional information, including other possible effects of cooling-induced sublethal sperm injury, is required to judge the cooling tolerance of boar ejaculates.

The high *in vitro* performance of cooled semen was confirmed in the subsequent field trial reported here, yielding equally high fertility results with semen stored at 5 °C compared to those conventionally stored at 17 °C. Consistent and well-defined AI conditions, together with a sufficiently high number of females are mandatory to assess the effects of semen treatment [18] in experiments evaluating this recently-introduced semen storage concept. The main difference in the current study from our previous insemination study [5] is that here oestrus detection and insemination were performed only once a day instead of twice daily, thus implementing the insemination management programme that is common practice in many herds worldwide. Compared to twice-daily oestrus detection, evaluation every 24 h evidently results in less predictable and often longer insemination-ovulation intervals, thereby necessitating longer survival times of functionally competent spermatozoa in the female sperm reservoirs. Cryopreservation of boar semen decreases sperm survival in the female reproductive tract to approximately 4 h compared to approximately 12 h in liquid semen stored at 17 °C [30]. It is anticipated that chilling and storage at 5 °C will also shorten the functional life span of spermatozoa due to sublethal chilling effects which might have remained undetected with the assays used here. A sufficient number of fully competent sperm in the semen doses are needed for maximum fertility [18]. Thus, a higher sperm number per dose (2.5 × 10^9^) was chosen compared to the conventional number (1.5 × 10^9^ [31]) in order to compensate for the additional challenges of once-daily oestrus detection and the employed insemination protocol. Noteworthy, the field trial conditions were highly standardised and factors with potential effects on fertility performance were strictly randomised in the groups to establish equal management conditions and to exclude sow-related bias. The present results clearly show that with the extender and the cooling rates used here, semen storage at 5 °C provides an applicable alternative to storage at 17 °C, either to prevent or to minimise resistance problems. Taking into account the high impact of farm conditions, genetics, insemination management and other non-semen related factors on fertility performance [18], there is certainly the need for further testing of hypothermic storage experiments on a larger scale on different farms with reduced numbers of spermatozoa per dose. Determining herd-specific lower limits of sperm numbers in the AI dose is particularly important for genetic and economic reasons.

The driving force to develop a practical hypothermic semen storage concept as part of pig reproduction management is to omit antibiotics in semen extenders. Delayed cooling is beneficial to sperm survival [32] but increases the risk of bacterial growth [20], especially if the bacterial load in the raw semen is high [6]. Similar to the results of our previous study [5], bacterial counts in the present *in vitro* study decreased from 2 × 10^4^ CFU/mL in raw semen to less than 10^3^ CFU/mL (maximum 773 CFU/mL) in antibiotic-free semen, reflecting an efficient bacteriostasis in hypothermically stored samples 24 h after semen extension. Even in samples supplemented with AB, up to 245 CFU/mL bacteria belonging to the *B. cepacia* complex were detected, a group of facultative pathogenic species with intrinsic resistance to gentamicin, that was not detected in the raw semen samples. These results highlight the permanent risk of contamination with existing (multi-)resistant bacteria during the processing of semen, as already reported in some AI station laboratories [9, 22]. It is to note that the initial diversity and distribution of Gram-positive and Gram-negative bacteria in raw semen were maintained in cooled samples, whereas the presence of antibiotics reduced the natural spectrum of bacteria to specific Gram-negative non-fermenters, thus favouring the survival of resistant bacteria.

The biological significance of bacterial counts and types for reproductive performance in pigs is under debate. In stored semen, the major concern is the loss of sperm viability and motility, accompanied by increased sperm agglutination [33]. However, sperm damage as a result of the presence of bacteria detected in boar semen becomes effective only at higher contamination rates than those observed in the present study, i.e. greater than × 10^6^ CFU/mL [9, 11, 34, 35]. Adverse effects on the sow’s genital tract by commensal or opportunistic bacteria, such as *E. coli, P. aeruginosa* or *Pasteurella* (Table S3), which originate from the boar’s reproductive tract, seems to be of minor concern because estrous sows have a low susceptibility to uterine inflammation [36]. Experimental insemination using 50 mL extended semen spiked with 10^7^–10^8^ CFU/mL of typical bacteria often found in boar ejaculates (E. coli, Staphylococcus sp., *Pseudomonas* sp.) led to regular fertility results and normal endometrial morphology [37]. Moreover, the present results also demonstrate a slight tendency that mesophilic species, e.g. *E. coli* and *Pasteurella,* rapidly decrease in numbers during hypothermic semen storage. From the opposite perspective, a potential physiological role played by semen microbes in the reproductive function is now being considered [38].

## Conclusion

Antibiotic-free preservation of boar semen at 5 °C is a potential alternative to conventional storage at 17 °C in the presence of AB. The low-temperature storage effectively inhibited the growth of bacteria in extended boar semen and maintained the original bacteria spectrum with a potential function in reproductive physiology. Noteworthy, strict hygiene measures and regular monitoring of animal health status are necessary to minimise the bacterial load in raw semen and to avoid the entry of specific pathogens in the AI doses. Subtle cold-shock effects detected on a single-cell basis were at a low level and did not influence fertility results when sufficient sperm numbers in the AI doses were used in a once-daily insemination routine. The performing of further trials with lower sperm numbers and under different farm conditions is encouraged to assess the efficiency of the proposed semen storage method. Altogether, the hypothermic storage concept provides a promising tool to counteract the increase in antibiotic resistance in the field of pig reproduction.

## Supplementary Information

**Additional file 1: Table S1.** Sublethal sperm damage in semen samples: Semen samples were extended in AndroStar® Premium and stored at 17 °C with antibiotics (17 °C w/AB; 0.25 g/L gentamicin sulphate) or at 5 °C without antibiotics (5 °C w/o AB): Distribution of viable (Yo-Pro-1 neg) spermatozoa (%; means ± SEM) with low (M540 negative) or high (M540 positive) membrane fluidity and intact (PNA negative) or defective (PNA positive) acrosome (*n* = 9 boars, experiment 1).

**Additional file 2: Table S2.** Sublethal damage of sperm function in semen samples: Semen samples were extended in AndroStar® Premium and stored at 17 °C with antibiotics (17 °C w/AB; 0.25 g/L gentamicin sulphate) or at 5 °C without antibiotics (5 °C w/o AB). After 72 h of storage, sublethal damage of sperm function were determined under capacitating (Tyrode A) and non-capacitating (Tyrode C) conditions: Distribution of viable (Hoechst 33258 negative) spermatozoa (%; means ± SEM) with high (JC-1 aggregate positive) or low (JC-1 aggregate negative) mitochondria membrane potential and high (Calbryte 630 positive) or low (Calbryte 630 negative) intracellular calcium (*n* = 9 boars, experiment 1).

**Additional file 3: Table S3.** Isolated bacteria species of potential concern for artificial insemination: Bacteria species were isolated from raw semen and split semen samples preserved in BTS with antibiotics (0.25 g/L gentamicin sulphate, BTS w/AB) at 17 °C or in AndroStar® Premium without antibiotics (APrem w/o AB) at 5 °C for 72 h (experiment 1).

**Additional file 4: Table S4.** Sperm motility and acrosome defects of semen pools (*n* = 6) used for insemination (experiment 2).

## Data Availability

The datasets generated in the current study are available from the corresponding author on reasonable request.
