# Supplementary material for: In vitro performance and in vivo fertility of antibiotic-free preserved boar semen stored at 5 °C
Source: J Anim Sci Biotechnol. 2021 Jan 11;12:9. doi: 10.1186/s40104-020-00530-6 (PMC7798330; doi:10.1186/s40104-020-00530-6)
Supplement: Supplementary file 1 — Additional file 1: Table S1. Sublethal sperm damage in semen samples: Semen samples were extended in AndroStar® Premium and stored at 17 °C with antibiotics (17 °C w/AB; 0.25 g/L gentamicin sulphate) or at 5 °C without antibiotics (5 °C w/o AB): Distribution of viable (Yo-Pro-1 neg) spermatozoa (%; means ± SEM) with low (M540 negative) or high (M540 positive) membrane fluidity and intact (PNA negative) or defective (PNA positive) acrosome (n = 9 boars, experiment 1). [file 40104_2020_530_MOESM1_ESM.pdf]

TABLE S1 Sublethal sperm damage in semen samples: Membrane fluidity in viable sperm (Experiment 1)

Semen storage		17 °C w/AB			5 °C w/o AB		
Fluorescence pattern		24 h	72 h	144 h	24 h	72 h	144 h
M540 neg	PNA neg	90.4 ± 2.3 $^{A, a}$	87.9 ± 3.1 $^{A, a}$	81.1 ± 4.1 $^{A, b}$	84.3 ± 4.4 $^{B, a}$	81.1 ± 5.7 $^{B, a}$	77.9 ± 5.1 $^{A, a}$
	PNA pos	3.1 ± 0.6 $^{A, a, b}$	2.9 ± 0.8 $^{A, a}$	4.1 ± 0.9 $^{A, b}$	2.8 ± 0.5 $^{A, a}$	2.5 ± 0.6 $^{B, a}$	2.8 ± 0.6 $^{A, a}$
M540 pos	PNA neg	5.6 ± 2.5 $^{A, a}$	8.1 ± 3.3 $^{A, a}$	13.5 ± 4.6 $^{A, b}$	11.7 ± 4.6 $^{B, a}$	15.2 ± 5.9 $^{B, b}$	18.0 ± 5.3 $^{A, a, b}$
	PNA pos	1.0 ± 0.2 ^{A}	1.0 ± 0.1 ^{A}	1.3 ± 0.1 ^{A}	1.2 ± 0.2 ^{B}	1.2 ± 0.2 ^{A}	1.3 ± 0.2 ^{A}

A-B) Values differ between storage temperatures within a given time point ($P < 0.05$)
a-b) Values differ between time points within a given storage temperature ($P < 0.05$)

Semen samples were extended in AndroStar® Premium and stored at 17 °C with antibiotics (17 °C w/AB; 0.25 g/L gentamicin sulphate) or at 5 °C without antibiotics (5 °C w/o AB): Distribution of viable (Yo-Pro-1 neg) spermatozoa (%; means ± SEM) with low (M540 negative) or high (M540 positive) membrane fluidity and intact (PNA negative) or defective (PNA positive) acrosome (n = 9 boars).
