# Supplementary material for: In vitro performance and in vivo fertility of antibiotic-free preserved boar semen stored at 5 °C
Source: J Anim Sci Biotechnol. 2021 Jan 11;12:9. doi: 10.1186/s40104-020-00530-6 (PMC7798330; doi:10.1186/s40104-020-00530-6)
Supplement: Supplementary file 2 — Additional file 2: Table S2. Sublethal damage of sperm function in semen samples: Semen samples were extended in AndroStar® Premium and stored at 17 °C with antibiotics (17 °C w/AB; 0.25 g/L gentamicin sulphate) or at 5 °C without antibiotics (5 °C w/o AB). After 72 h of storage, sublethal damage of sperm function were determined under capacitating (Tyrode A) and non-capacitating (Tyrode C) conditions: Distribution of viable (Hoechst 33258 negative) spermatozoa (%; means ± SEM) with high (JC-1 aggregate positive) or low (JC-1 aggregate negative) mitochondria membrane potential and high (Calbryte 630 positive) or low (Calbryte 630 negative) intracellular calcium (n = 9 boars, experiment 1). [file 40104_2020_530_MOESM2_ESM.pdf]

TABLE S2 Sublethal damage of sperm function: Mitochondria membrane potential and calcium content during capacitation (Experiment 1)

		17 °C w/AB				5 °C w/o AB			
		Tyrode A		Tyrode C		Tyrode A		Tyrode C	
Fluorescence pattern		3 min	60 min	3 min	60 min	3 min	60 min	3 min	60 min
JC-1 agg neg	Calbryte neg	2.8 ± 0.8 [a]	2.1 ± 0.6 [a]	2.8 ± 0.8 [a]	2.3 ± 0.8 [b]	1.8 ± 0.4 [a]	1.4 ± 0.2 [b]	1.7 ± 0.4 [a]	1.1 ± 0.2 [b]
	Calbryte pos	6.6 ± 1.0 [a]	18.0 ± 3.9 [b]	7.3 ± 1.0 [a]	8.3 ± 1.2 [b]	7.0 ± 1.0 [a]	18.8 ± 2.6 [b]	7.0 ± 1.1 [a]	10.0 ± 1.3 [b]
JC-1 agg pos	Calbryte neg	79.3 ± 1.2 [a]	49.6 ± 5.6 [b]	78.2 ± 0.9 [a]	73.3 ± 2.0 [A, b]	79.3 ± 1.0 [a]	44.0 ± 3.9 [b]	78.6 ± 1.1 [a]	66.3 ± 2.5 [B, b]
	Calbryte pos	11.3 ± 1.0 [a]	30.3 ± 4.0 [b]	11.7 ± 1.3 [a]	16.1 ± 2.1 [A, b]	11.9 ± 0.6 [a]	35.8 ± 2.5 [b]	12.6 ± 0.8 [a]	22.6 ± 1.8 [B, b]

A-B) Values differ between storage temperatures within a given time point and medium ($P < 0.05$)

a-b) Values differ between time points within a given storage temperature and medium ($P < 0.05$)

Semen samples were extended in AndroStar® Premium and stored at 17 °C with antibiotics (17 °C w/AB; 0.25 g/L gentamicin sulphate) or at 5 °C without antibiotics (5 °C w/o AB). After 72 h of storage, sublethal damages of sperm function were determined under capacitating (Tyrode A) and non-capacitating (Tyrode C) conditions: Distribution of viable (Hoechst 33258 negative) spermatozoa (%; means ± SEM) with high (JC-1 aggregate positive) or low (JC-1 aggregate negative) mitochondria membrane potential and high (Calbryte 630 positive) or low (Calbryte 630 negative) intracellular calcium (n = 9 boars).
