# Supplementary material for: In vitro performance and in vivo fertility of antibiotic-free preserved boar semen stored at 5 °C
Source: J Anim Sci Biotechnol. 2021 Jan 11;12:9. doi: 10.1186/s40104-020-00530-6 (PMC7798330; doi:10.1186/s40104-020-00530-6)
Supplement: Supplementary file 3 — Additional file 3: Table S3. Isolated bacteria species of potential concern for artificial insemination: Bacteria species were isolated from raw semen and split semen samples preserved in BTS with antibiotics (0.25 g/L gentamicin sulphate, BTS w/AB) at 17 °C or in AndroStar® Premium without antibiotics (APrem w/o AB) at 5 °C for 72 h (experiment 1). [file 40104_2020_530_MOESM3_ESM.pdf]

TABLE S3: Isolated bacteria species of potential concern for artificial insemination (Experiment 1)

**Bacteria species**	**Detection at storage time**	**Type of semen sample (n = 9 boars)**		
		**Raw semen, n**	**17 °C BTS w/AB, n**	**5 °C APrem w/o AB, n**
Burkholderia cepacia complex	0 to 72 h	0	6	9
Pseudomonas aeruginosa	0 to 72 h	4	0	6
Pasteurella sp.	0 to 24 h	1	0	2
Escherichia coli	0 h	1	0	1

Bacteria species were isolated from raw semen and extended semen samples preserved in Beltsville Thawing Solution with antibiotics (0.25 g/L gentamicin sulphate, BTS w/AB) at 17 °C or in AndroStar® Premium without antibiotics (APrem w/o AB) at 5 °C for 72 h. Maximal bacterial counts were 245 CFU/mL in BTS w/AB and 773 CFU/mL in APrem w/o AB.
