# Supplementary material for: In vitro performance and in vivo fertility of antibiotic-free preserved boar semen stored at 5 °C
Source: J Anim Sci Biotechnol. 2021 Jan 11;12:9. doi: 10.1186/s40104-020-00530-6 (PMC7798330; doi:10.1186/s40104-020-00530-6)
Supplement: Supplementary file 4 — Additional file 4: Table S4. Sperm motility and acrosome defects of semen pools (n = 6) used for insemination (experiment 2). [file 40104_2020_530_MOESM4_ESM.pdf]

TABLE S4: Sperm motility and acrosome defects of semen pools (n = 6) used for insemination (Experiment 2)

Semen sample	17 °C w/AB			5 °C w/o AB		
**Storage time**	**24 h**	**72 h**	**120 h**	**24 h**	**72 h**	**120 h**
**Total motility, %**	92.7 ± 0.5 ^{A}	93.0 ± 0.9 ^{A}	93.0 ± 0.5 ^{A}	90.0 ± 1.0 ^{A}	88.5 ± 1.0 ^{B}	88.9 ± 0.6 ^{B}
**Defective acrosomes, %**	2.6 ± 0.3 $^{A, a}$	2.6 ± 0.3 $^{A, a}$	4.3 ± 0.4 $^{A, b}$	2.5 ± 0.1 $^{A, a}$	4.2 ± 0.5 $^{B, b}$	4.7 ± 0.4 $^{A, b}$

17 °C: semen stored in AndroStar® Premium with antibiotics (w/AB; 0.25 g/L gentamicin sulphate)
5 °C: semen stored in AndroStar® Premium without antibiotics (w/o AB)
Values are shown as means ± SEM

A-B) Values differ between storage temperatures within a given time point ($P < 0.05$)
a-b) Values differ between time points within a given storage temperature ($P < 0.05$)
